# Histological Insights into the Neuroprotective Effects of Antioxidant Peptides and Small Molecules in Cerebral Ischemia

**DOI:** 10.3390/molecules30234529

**Published:** 2025-11-24

**Authors:** Sanda Jurja, Ticuta Negreanu-Pirjol, Mihaela Cezarina Mehedinți, Maria-Andrada Hincu, Anca Cristina Lepadatu, Bogdan-Stefan Negreanu-Pirjol

**Affiliations:** 1 Faculty of Medicine, “Ovidius” University of Constanta, 1, University Alley, Campus Building B, 900470 Constanta, Romania; sanda.jurja@365.univ-ovidius.ro; 2 Faculty of Pharmacy, “Ovidius” University of Constanta, 6, Capitan Aviator Al. Serbanescu Street, Campus Building C, 900470 Constanta, Romania; bogdan.negreanu@univ-ovidius.ro; 3 Academy of Romanian Scientists, Biological Sciences Section, 3, Ilfov Street, 50044 Bucharest, Romania; 4 Faculty of Medicine, “Dunarea de Jos” University of Galati, 800712 Galati, Romania; mariaandradaa99@gmail.com; 5 Faculty of Natural Sciences and Agricultural Sciences, “Ovidius” University of Constanta, 1, University Alley, Campus Building B, 900470 Constanta, Romania; anca.lepadatu@365.univ-ovidius.ro

**Keywords:** cerebral ischemia, ischemic stroke, oxidative stress, neuroprotection, antioxidant peptides, small-molecule antioxidants, histology, ferroptosis, apoptosis

## Abstract

Cerebral ischemia represents a major mortality and disability cause; oxidative stress is the main intensifier mechanism of excitotoxicity, neuroinflammation, blood–brain barrier failure, and neuronal loss; under these circumstances, firm, mechanism-anchored neuroprotection is an absolute necessity. The work includes a exhaustive, PRISMA (Preferred reporting items for systematic review and meta-analysis)-adherent presentation of the effects of antioxidant peptides and small molecules on tissues, unifying disparate readouts into a coherent tissue-level narrative. A systematic interrogation was performed across major databases over a prespecified interval, applying transparent eligibility criteria to studies that quantified canonical endpoints—infarct volume, neuronal integrity (NeuN/MAP2), apoptosis (TUNEL/cleaved caspase-3), gliosis (GFAP/Iba1), and ultrastructural preservation. The evidence coalesces around a strikingly consistent signal: antioxidant strategies converge on smaller infarcts, robust preservation of neuronal markers, attenuation of apoptotic burden, dampened astroglial–microglial reactivity, and stabilization of mitochondrial and axonal architecture—patterns that align with antioxidative, anti-apoptotic, anti-inflammatory, and ferroptosis-modulating mechanisms. While early clinical data echo these benefits, translation is tempered by heterogeneity in models, timing and dosing windows, and outcome batteries. By consolidating the histological landscape and pinpointing where effects are durable versus contingent, this work elevates antioxidant peptide and small-molecule neuroprotection from promising fragments to an integrated framework and sets an actionable agenda—standardized histological endpoints, protocol harmonization, head-to-head comparisons of peptide versus small-molecule strategies, and adequately powered randomized trials embedded with mechanistic biomarkers to decisively test efficacy and accelerate clinical adoption.

## 1. Discovery Phase

### 1.1. Prevalence and Impact of Cerebral Ischemia

Cerebral ischemia remains a dominant cause of mortality and disability, with oxidative stress acting as the principal amplifier of excitotoxicity, neuroinflammation, blood–brain barrier failure, and neuronal loss. Accordingly, rigorous, mechanism-anchored neuroprotection is a pressing need. This review provides a comprehensive, PRISMA-adherent synthesis of the histological effects of antioxidant peptides and small molecules, unifying disparate readouts into a coherent tissue-level narrative. We systematically investigated major databases over a prespecified interval, applying transparent eligibility criteria to studies that quantified canonical endpoints—infarct volume, neuronal integrity (NeuN/MAP2), apoptosis (TUNEL/cleaved caspase-3), gliosis (GFAP/Iba1), and ultrastructural preservation. The evidence coalesces around a strikingly consistent signal: antioxidant strategies converge on smaller infarcts, robust preservation of neuronal markers, attenuation of apoptotic burden, dampened astroglial–microglial reactivity, and stabilization of mitochondrial and axonal architecture—patterns that align with antioxidative, anti-apoptotic, anti-inflammatory, and ferroptosis-modulating mechanisms. While early clinical data echo these benefits, translation is tempered by heterogeneity in models, timing and dosing windows, and outcome measures. By consolidating the histological landscape and pinpointing where effects are durable versus contingent, this study elevates antioxidant peptide and small-molecule neuroprotection from promising fragments to an integrated framework and sets an actionable agenda: standardized histological endpoints, protocol harmonization, head-to-head comparisons of peptide versus small-molecule strategies, and adequately powered randomized trials embedded with mechanistic biomarkers to decisively test efficacy and accelerate clinical adoption.

### 1.2. Role of Oxidative Stress in Pathogenesis

The ischemic cascade is initiated by the abrupt cessation of cerebral blood flow and energy failure, which collectively set excitotoxicity, ionic imbalance, mitochondrial dysfunction, inflammation, and cell death into motion. Among these interconnected processes, oxidative stress is consistently recognized as a unifying and amplifying mechanism. The excessive generation of reactive oxygen species (ROS) during ischemia and subsequent reperfusion results in widespread lipid peroxidation, protein misfolding, and genomic instability, thereby driving neuronal apoptosis and necrosis [1,2,3].

Large-scale and high-impact studies have reinforced this view by demonstrating that dysregulated redox signaling profoundly alters neuronal survival pathways, endothelial integrity, and immune cell activation [2,4]. Such findings highlight that oxidative stress not only mediates direct cytotoxicity but also acts as a central hub that links metabolic failure to inflammation and blood–brain barrier disruption. This dual role—as both an initiator and amplifier of injury—explains why targeting oxidative stress has become a major therapeutic strategy in experimental stroke research.

By positioning oxidative stress at the intersection of multiple damaging cascades, the literature underscores its importance as a critical therapeutic target. This perspective provides the conceptual foundation for evaluating antioxidant peptides and small molecules as adjunctive neuroprotective strategies in ischemic stroke.

In addition to direct neuronal toxicity, oxidative stress also exacerbates neuroinflammatory signaling and disrupts the blood–brain barrier, resulting in secondary injury and impaired recovery [5]. Oxidative stress emerges not merely as a hallmark but as an active driver of ischemic brain damage, a role that strengthens the rationale for antioxidant-based therapeutic interventions.

### 1.3. Rationale for the Use of Antioxidant Peptides and Small Molecules

Given the central role of redox imbalance in the ischemic cascade, targeting oxidative stress has emerged as a major therapeutic focus. Antioxidant peptides constitute a promising class of neuroprotective agents due to their multifaceted mechanisms of action, including free radical scavenging, modulation of apoptotic pathways, and reinforcement of endogenous antioxidant defenses. Notably, peptidomimetic inhibitors designed to disrupt PSD-95/nNOS interactions have exhibited robust neuroprotection capabilities both in vitro and in vivo. In transient middle cerebral artery occlusion (MCAO) models, these agents not only reduced infarct volume but also attenuated neuronal degeneration, as confirmed via Nissl staining and histological assessments of cortical and striatal regions [6].

In parallel, small molecules—defined in pharmacological sciences as low-molecular-weight organic compounds (typically < 900 Da) capable of modulating biological processes by interacting with specific proteins, enzymes, or nucleic acids—have been extensively investigated as antioxidant therapies. Their relatively small size confers favorable pharmacokinetic properties, such as oral bioavailability and blood–brain barrier permeability, which make them attractive candidates for translational applications. Gastrodin, a naturally occurring small molecule, promoted Alkbh5 nuclear localization and mitigated oxidative stress in ischemic stroke models. Histological analyses revealed reduced neuronal apoptosis, demonstrated by decreased TUNEL-positive cells, alongside improved preservation of NeuN-positive neurons, indicating direct structural protection [7]. Similarly, remote ischemic preconditioning, although not a small molecule in itself, effectively enhanced antioxidant capacity and attenuated oxidative damage. Importantly, this intervention produced measurable histological benefits, including reduced neuronal loss and diminished cerebral edema in experimental stroke models [4].

Antioxidant peptides and small molecules exhibit neuroprotection through dual mechanisms: restoration of redox balance with consequent reduction in ROS and preservation of neuronal and tissue architecture as observed histologically. This integrated action provides mechanistic plausibility for their use in combination with reperfusion therapies.

### 1.4. Objectives of the Review

This systematic review aims to provide an overview of the available evidence on histological insights into the neuroprotective effects of antioxidant peptides and small molecules in cerebral ischemia. Specifically, the objectives are as follows:

1. To summarize histological outcomes (neuronal survival, apoptosis markers, and structural preservation) associated with these compounds.
2. To correlate histological evidence with the biochemical markers of oxidative stress and functional outcomes.
3. To compare the relative contributions of peptide-based versus small-molecule antioxidants.
4. To identify gaps in the literature and highlight opportunities for translational and clinical research.

## 2. Analytical Framework

### 2.1. Protocol and Registration

This systematic review was conducted in accordance with the Preferred Reporting Items for Systematic Reviews and Meta-Analyses (PRISMA 2020) recommendations.

The prespecified protocol detailed the PICOS framework (population, intervention, comparator, outcomes, and study design), eligibility criteria, information sources and search strategy, study selection workflow, data extraction items, and risk-of-bias assessment tools (SYRCLE for animal studies; Cochrane risk of bias for clinical studies), as well as the qualitative synthesis plan. Any deviations from the registered protocol were transparently documented and justified in this manuscript and reflected in the PROSPERO record.

### 2.2. Eligibility Criteria (PICO Framework)

With respect to the study population, the inclusion criteria comprised the following: adult patients (≥18 years) with cerebral ischemia or ischemic stroke confirmed via clinical and/or imaging criteria; and patients reporting histological outcomes, such as neuronal survival, infarct size, apoptosis, oxidative stress, or neuroinflammation. Eligible study designs include randomized controlled trials (RCTs), cohort studies, and case–control studies, as well as translational animal models of cerebral ischemia that incorporate histological endpoints. The exclusion criteria were as follows: pediatric populations (<18 years), hemorrhagic or mixed-type strokes without ischemia-specific outcomes, studies lacking histological data, case reports, editorials, reviews, and conference abstracts without sufficient methodological detail.

With respect to interventions, the inclusion criteria were as follows: antioxidant peptides and small-molecule antioxidants evaluated for neuroprotective potential in cerebral ischemia. Eligible peptides include endogenous compounds (e.g., glutathione, carnosine) and synthetic or engineered peptides (e.g., SS-31/elamipretide, OL-FS13). Small molecules include established antioxidants such as melatonin, resveratrol, curcumin, and edaravone, as well as other pharmacological agents with demonstrated antioxidant and neuroprotective activity. All modes of administration (oral, intravenous, intraperitoneal, and intranasal) and dosing regimens are eligible, provided that histological outcomes are reported. Interventions may be tested alone or in combination with standard therapies, but the antioxidant-specific effect must be distinguishable. The exclusion criteria were as follows: Interventions without clearly defined antioxidant peptides or small molecules; dietary supplements, complex nutraceutical mixtures, or lifestyle-based antioxidant strategies (e.g., diet, exercise) without a specific compound; multimodal pharmacological combinations where the independent effect of the antioxidant cannot be isolated; and studies exclusively reporting biochemical, imaging, or behavioral outcomes without histological assessments. Interventions applied in non-ischemic contexts (e.g., traumatic brain injury, neurodegeneration) are also excluded.

With respect to comparators, the inclusion criteria were as follows: placebo, sham treatment, no intervention, or standard of care/usual medical management for cerebral ischemia. Head-to-head comparisons between different antioxidant peptides or small molecules are also eligible, provided that histological outcomes are reported. The exclusion criteria were as follows: studies with poorly defined or historical controls and comparators involving complex multimodal interventions in which the antioxidant-specific effect cannot be isolated. Non-specific lifestyle or supportive care comparators without a pharmacological reference arm are not eligible.

Outcomes: The primary outcome is histological evidence of neuroprotection, including but not limited to reductions in infarct volume, increased neuronal survival, and decreased apoptosis (as assessed via Nissl staining, NeuN immunoreactivity, TUNEL assay, H&E, GFAP, MAP2, or related histological methods). Secondary outcomes include markers of oxidative stress, neuroinflammation, blood–brain barrier integrity, and functional recovery when correlated with histological endpoints.

With respect to study design, the inclusion criteria were as follows: Randomized controlled trials are prioritized for methodological rigor. Non-randomized clinical studies (prospective or retrospective cohorts; case–control studies) are also eligible if histological endpoints are reported. Translational preclinical studies using animal models of cerebral ischemia (e.g., middle cerebral artery occlusion, global ischemia) are included if histological outcomes are systematically assessed. Both acute and subacute models are considered, provided that standard histological or immunohistochemical methods are used. The exclusion criteria were as follows: case reports, case series without comparators, conference abstracts without sufficient data, editorials, commentaries, and narrative reviews.

Context: Eligible studies include clinical trials conducted in acute care or neurology departments, stroke units, and rehabilitation centers, provided that histological or neuropsychological outcomes are reported. Both single-center and multicenter studies are eligible, with no geographic restrictions. Preclinical studies must be conducted in controlled laboratory environments, including well-established experimental models of ischemia, and they must include validated histological outcomes. No restrictions were applied regarding country, healthcare system, or economic setting.

### 2.3. Search Strategy

A systematic search was undertaken in three electronic databases: PubMed/MEDLINE, Scopus, and the Web of Science Core Collection. The search covered the period from January 2020 to March 2025 and was designed to capture both clinical and experimental studies investigating antioxidant peptides and small-molecule antioxidants in cerebral ischemia.

The strategy combined controlled vocabulary (e.g., MeSH terms in PubMed) with free-text keywords, using Boolean operators (AND, OR) to maximize both sensitivity and specificity. Three main concepts were incorporated: (i) the condition of interest (cerebral ischemia and ischemic stroke), (ii) interventions (antioxidant peptides, peptidomimetics, and small molecules), and (iii) outcomes (histology, Nissl, TUNEL, NeuN, and neuroprotection).

For example, the PubMed query included terms such as “cerebral ischemia” and “ischemic stroke”, combined with “antioxidant peptides” OR “peptidomimetics” OR “small molecules”, and outcome-related keywords (“histology”, “Nissl”, “TUNEL”, “NeuN”, and “neuroprotection”). Equivalent search strategies were adapted for Scopus and Web of Science using the same Boolean structure and temporal filters.

The final search, last updated on 31 March 2025, yielded a total of 1819 records: 812 from PubMed, 673 from Scopus, and 334 from Web of Science. These results were exported, deduplicated, and screened in accordance with the PRISMA 2020 guidelines.

### 2.4. Data Extraction

Data from all studies meeting the inclusion criteria were extracted independently by two reviewers using a predesigned template. Discrepancies in extracted data were resolved by consensus or adjudicated by a third reviewer. The complete dataset was subsequently used to construct evidence tables summarizing interventions, experimental models, and histological outcomes.

**Figure 1:**
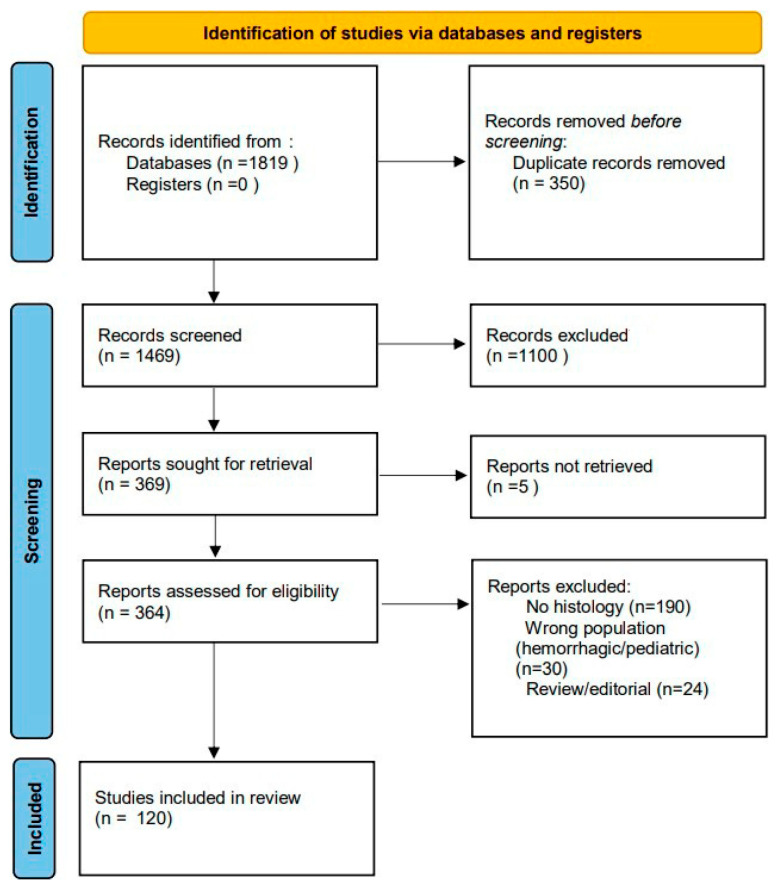
PRISMA 2020 flow diagram illustrating the study selection process. From a total of 1819 records initially identified, after duplicate removal, screening, and eligibility assessment, 120 studies were included in the final qualitative synthesis.

Across all three databases, the search yielded a total of 1819 records before deduplication. After automatic and manual duplicate removal, unique records were taken forward for title/abstract screening in accordance with the PRISMA workflow. To ensure transparency of the selection process, the number of records identified, screened, assessed for eligibility, and included in the qualitative synthesis is summarized in the PRISMA 2020 flow diagram (Figure 1). This diagram details the progression from the initial 1819 records retrieved across databases to the final 120 studies that met the prespecified inclusion criteria.

## 3. Molecular and Histological Pathophysiology of Cerebral Ischemia

Cerebral ischemia initiates a multifactorial cascade within minutes of vascular occlusion and evolves into complex secondary injury over hours to days. At the molecular level, oxygen–glucose deprivation precipitates a failure of oxidative phosphorylation with a collapse of mitochondrial respiratory function and ATP generation, as demonstrated by impaired oxygen consumption and loss of mitochondrial membrane potential in neurons and brain microvascular endothelial cells after OGD/R, and by the hemisphere-specific suppression of electron transport chain activity with elevated ROS emissions after MCAO [8,9]. Consequent redox disequilibrium manifests as excessive reactive oxygen species (ROS) production and the activation of oxidative damage pathways that propagate tissue injury [8,9]. Excessive ROS initiate lipid peroxidation, carbonylation of structural proteins, and fragmentation of nuclear DNA, creating conditions conducive to both apoptotic and ferroptotic forms of cell death [10]. Recent evidence indicates that ferroptosis, an iron-dependent program of lipid peroxide accumulation, plays a particularly prominent role in ischemia–reperfusion injury, with histological correlates including swollen or shrunken mitochondria, condensed chromatin, and cytoplasmic vacuolization. Interventions that suppress ferroptosis, such as wedelolactone, have been shown to reduce infarct size and improve neuronal preservation by attenuating both ROS production and ferroptotic signaling cascades [10].

Concomitant excitotoxicity exacerbates oxidative injury. Following ischemia, excessive extracellular glutamate overstimulates NMDA and AMPA receptors, causing calcium influx and mitochondrial overload. The ensuing mitochondrial collapse enhances free radical generation and activates caspase-mediated apoptosis. These mechanisms are consistently reflected in histological studies, which show decreased NeuN+ neuronal density, reduced MAP2+ dendritic labeling, and increased TUNEL-positive nuclei in ischemic cortices and hippocampi. Natural compounds such as gastrodin mitigate these deleterious changes, reducing apoptosis and preserving neuronal morphology, thereby supporting the mechanistic role of oxidative and excitotoxic injury in histological tissue loss [7]. Similarly, conditioning paradigms such as remote ischemic preconditioning strengthen endogenous antioxidant defenses and promote neuronal survival, reducing apoptotic signatures and supporting long-term structural integrity [4].

A third axis of the ischemic cascade is neuroinflammation, which develops within hours and persists into subacute and chronic phases. Activated microglia release inflammatory cytokines and reactive nitrogen species, further amplifying oxidative stress. Astrocytic proliferation (GFAP+) contributes to glial scarring, while endothelial dysfunction compromises the blood–brain barrier’s (BBB) integrity, allowing the infiltration of peripheral immune cells. Histological studies consistently demonstrate increased GFAP+ astrogliosis, Iba1+ microglial activation, and perivascular edema in untreated ischemic models [11]. Pharmacological approaches that directly target neuroinflammation are emerging as highly promising. For instance, the novel compound CP-10 suppresses microglial activation and downstream inflammatory signaling, which correlates histologically with reduced gliosis, improved MAP2+ dendritic preservation, and diminished neuronal apoptosis [11].

Additional research has highlighted the importance of nuclear regulatory pathways and epigenetic modulators in shaping ischemic injury. Gastrodin, for example, influences Alkbh5 nuclear localization, which reduces pro-apoptotic signaling and contributes to improved neuronal survival [7]. These data extend the mechanistic landscape beyond ROS scavenging to include the regulation of transcriptional responses, post-translational modifications, and mitochondrial–nuclear communication. The histological correlates of these interventions are remarkably consistent: reduced infarct size, preservation of NeuN+ neuronal morphology, attenuated TUNEL positivity, and decreased GFAP+ astrogliosis.

The body of evidence published since 2020 delineates a tightly interconnected model of cerebral ischemia pathophysiology. Oxidative stress and ferroptosis emerge as initiating molecular insults, excitotoxicity acts as a powerful amplifier, and neuroinflammation perpetuates and expands tissue damage. These cascades are consistently reflected in histological signatures of neuronal death, glial activation, and mitochondrial disruption, providing reliable outcome measures for therapeutic evaluation. Convergent findings across natural compounds, synthetic peptides, and conditioning paradigms reinforce the centrality of oxidative stress and supply a strong mechanistic rationale for antioxidant- and anti-inflammatory-based interventions.

## 4. Empirical Evidence

### 4.1. Data Synthesis and Rationale for Tabular Presentation

Given the heterogeneity of the included studies in terms of molecular interventions, experimental models, and reported outcomes, a structured synthesis was necessary to enable meaningful comparisons. A large proportion of the evidence base consisted of preclinical experiments employing focal cerebral ischemia models, most commonly the middle cerebral artery occlusion (MCAO) technique in rodents. This model remains the gold standard for mimicking human ischemic stroke, as it closely reproduces the cascade of excitotoxicity, oxidative stress, inflammation, and neuronal death observed in patients. In addition, a limited number of clinical trials and pilot studies were identified, primarily evaluating well-established antioxidant compounds such as edaravone and melatonin.

The synthesis presented in Table 1 provides a structured overview of the evidence base for antioxidant peptides and small molecules in cerebral ischemia, emphasizing both the quantity of available studies and the spectrum of histological outcomes reported. The distribution of evidence reveals that the majority of compounds have been tested primarily in preclinical models, particularly in middle cerebral artery occlusion (MCAO) paradigms, where histological endpoints such as infarct volume, neuronal survival, and apoptosis have been systematically assessed [12]. A smaller but noteworthy subset of agents, most prominently melatonin and edaravone, has progressed to clinical testing, underscoring their translational potential and the maturity of the supporting evidence base [13].

A closer examination of the molecules indicates that some, such as triptolide, quercetin, and curcumin, show consistent histological benefits in animal studies, yet they lack validation in human cohorts [12]. Others, such as resveratrol (5-[(E)-2-(4-hydroxyphenyl)ethenyl]benzene-1,3-diol) and elamipretide (H-D-Arg-Tyr(2,6-diMe)-Lys-Phe-NH2), demonstrate promising preclinical signals, including the attenuation of gliosis, preservation of neuronal markers (NeuN and MAP2), and reduction in oxidative stress, but they remain underrepresented in clinical trials. The inclusion of the *other small molecules*/*mixed antioxidants* category reflects the methodological necessity of grouping agents tested in isolated or heterogeneous contexts, thereby maintaining completeness while avoiding over-fragmentation.

**Figure 2:**
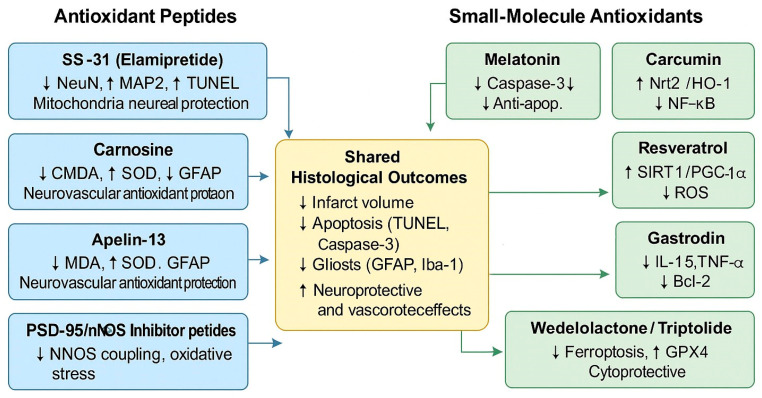
Representative antioxidant peptides and small molecules investigated in cerebral ischemia and their shared histological outcomes. Arrows show increasing↑/decreasing↑ trend.

Evidence accumulated to date emphasizes a dual reality. On the one hand, animal studies have defined oxidative stress and related pathways as central drivers of neuronal injury, with consistent histological readouts across laboratories. On the other hand, the paucity of large clinical trials underscores how limited the translational step has been. Against this background, the next sections provide a molecule-by-molecule analysis, focusing on histological consistency, mechanistic depth, and opportunities for clinical translation in ischemic stroke therapy. Peptides such as SS-31 (elamipretide), carnosine, apelin-13, and PSD-95/nNOS inhibitor peptides primarily target mitochondrial stability and oxidative stress reduction, while small molecules, including melatonin, curcumin, resveratrol, gastrodin, wedelolactone, and triptolide, exert synergistic antioxidant, anti-inflammatory, and anti-apoptotic effects. Collectively, these interventions contribute to decreased infarct volume, apoptosis, and gliosis, as well as enhanced neuronal preservation (NeuN and MAP2 expression) and neurovascular protection, as presented in Figure 2.

### 4.2. Antioxidant Peptides as Neuroprotective Agents

Antioxidant peptides have emerged as a distinct class of neuroprotective agents in cerebral ischemia, offering advantages related to their intrinsic radical-scavenging capacity, ability to modulate cell death pathways, and favorable biocompatibility [14]. Several endogenous and synthetic peptides have been tested in preclinical ischemia models, consistently demonstrating histological benefits, such as reductions in infarct volume, attenuation of apoptosis, and preservation of neuronal morphology. For example, elamipretide (SS-31), a mitochondria-targeted tetrapeptide, significantly improved NeuN+ neuronal survival and reduced oxidative stress markers in middle cerebral artery occlusion (MCAO) models [15,16]. Similarly, carnosine, a naturally occurring dipeptide, reduced infarct volume and enhanced neuronal survival, effects associated with decreased ROS accumulation and the inhibition of caspase-mediated apoptosis [17,18]. Apelin-13, another small regulatory peptide, not only diminished infarct size but also improved NeuN+ immunoreactivity, indicating enhanced neuronal preservation in ischemic penumbra [19,20].

Additional evidence supports the protective role of oligopeptides such as OL-FS13, which suppressed microglial activation and preserved MAP2+ dendritic integrity, thereby limiting neuroinflammation and secondary histological damage [21]. Peptide-based interventions exhibit a broad histological profile of neuroprotection, encompassing the acute suppression of oxidative stress and apoptosis, as well as the sustained preservation of neuronal and glial architecture. However, despite the consistency of these preclinical findings, clinical translation remains limited, and agents such as SS-31 and carnosine have not advanced beyond experimental models. This gap in research underscores the need for rigorously designed translational studies to determine whether the histological benefits observed in animal models can be replicated in human ischemic stroke patients. Beyond these well-characterized compounds, other experimental antioxidant peptides have shown promising results in ischemia models. For instance, humanin (H-Met-Ala-Pro-Arg-Gly-Phe-Ser-Cys-Leu-Leu-Leu-Leu-Thr-Ser-Glu-Ile-Asp-Leu-Pro-Val-Lys-Arg-Arg-Ala-OH), a mitochondria-associated peptide, has been reported to preserve neuronal ultrastructure and reduce TUNEL+ apoptotic cell counts in transient MCAO paradigms, with concurrent reductions in lipid peroxidation markers and the restoration of mitochondrial respiratory enzyme activity [22]. Likewise, thymosin-β4 exerted significant histological benefits by attenuating glial scar formation, reducing GFAP+ astrocytic proliferation, and enhancing NeuN+ neuronal density in peri-infarct regions [23]. These findings extend the neuroprotective spectrum of antioxidant peptides to encompass both neuronal and glial compartments, suggesting pleiotropic modes of action.

Another innovative approach involves engineered peptidomimetics that selectively target protein–protein interactions involved in excitotoxic and oxidative pathways. The peptidic inhibitors of PSD-95/nNOS coupling, for example, reduced infarct volume and improved neuronal survival in MCAO models by simultaneously suppressing nitric oxide overproduction and downstream oxidative injury [24]. Histological analyses revealed reduced neuronal apoptosis and improved preservation of cortical lamination, reinforcing the therapeutic potential of rationally designed antioxidant peptides.

Taken together, these diverse studies indicate that antioxidant peptides act through complementary mechanisms, including direct radical scavenging, mitochondrial stabilization, anti-apoptotic signaling, and attenuation of neuroinflammation [25]. The reproducibility of histological endpoints—such as reduction in infarct size, increased NeuN and MAP2 immunoreactivity, decreased TUNEL positivity, and modulation of gliosis—underscores their translational promise. Nevertheless, the lack of robust clinical evaluation remains a critical limitation. While certain peptides, such as carnosine and SS-31, have entered early safety testing, large-scale randomized controlled trials are still absent. Bridging this translational gap will require systematic efforts to standardize dosing regimens, administration routes, and histological outcome measures in both preclinical and clinical settings. Antioxidant peptides have emerged as a distinct class of neuroprotective agents in cerebral ischemia, offering advantages related to their intrinsic radical-scavenging capacity, ability to modulate cell death pathways, and favorable biocompatibility. Several endogenous and synthetic peptides have been tested in preclinical ischemia models, consistently demonstrating histological benefits, such as reductions in infarct volume, attenuation of apoptosis, and preservation of neuronal morphology. For example, elamipretide (SS-31), a mitochondrion-targeted tetrapeptide, significantly improved NeuN+ neuronal survival and reduced oxidative stress markers in middle cerebral artery occlusion (MCAO) models [15,16]. Similarly, carnosine, a naturally occurring dipeptide, reduced infarct volume and enhanced neuronal survival, effects associated with decreased ROS accumulation and the inhibition of caspase-mediated apoptosis [17,18]. Apelin-13, another small regulatory peptide, not only diminished infarct size but also improved NeuN+ immunoreactivity, indicating enhanced neuronal preservation in ischemic penumbra [19,20]. Additional evidence supports the protective role of oligopeptides such as OL-FS13, which suppressed microglial activation and preserved MAP2+ dendritic integrity, thereby limiting neuroinflammation and secondary histological damage [21]. Collectively, these findings highlight the broad histological spectrum of peptide-mediated neuroprotection, ranging from acute effects on oxidative stress and apoptosis to longer-term structural preservation. However, despite robust preclinical data, clinical translation remains scarce, with most peptides—including SS-31 and carnosine—yet to progress beyond experimental models. This gap underscores the need for rigorously designed translational studies to determine whether the histological benefits observed in animal models can be replicated in human ischemic stroke patients. Beyond these well-characterized compounds, other studies to date highlight the diverse mechanistic repertoire of antioxidant peptides, encompassing mitochondrial protection, direct ROS scavenging, anti-apoptotic signaling, and the modulation of gliosis. To distill these observations and emphasize the histological endpoints consistently reported across experimental paradigms, Table 2 provides a structured overview of the principal peptides, their experimental models, mechanisms of action, and histological outcomes.

### 4.3. Small-Molecule Antioxidants

A broad spectrum of small-molecule antioxidants has been investigated as experimental therapeutics for ischemic stroke, with particular emphasis on their capacity to mitigate ferroptosis, oxidative stress, and secondary injury cascades. Importantly, their efficacy is not confined to biochemical markers but is consistently corroborated by *histological* evidence, demonstrating preserved cytoarchitecture, reduced infarct volume, and attenuated neuroinflammation.

#### 4.3.1. Melatonin

Melatonin is one of the most extensively studied small-molecule antioxidants in cerebral ischemia/reperfusion injury. Beyond its well-documented ability to scavenge free radicals and modulate iron-dependent lipid peroxidation, histological studies have consistently demonstrated robust structural protection. In MCAO models, melatonin treatment significantly decreased infarct size, preserved cortical layering, and prevented neuronal shrinkage. Nissl staining revealed a higher density of intact neurons in the ischemic penumbra, while hematoxylin–eosin (H&E) sections showed reduced cytoplasmic vacuolization and nuclear pyknosis [25,26]. These findings indicate that melatonin not only delays ferroptotic pathways but also results in the measurable preservation of brain histology.

#### 4.3.2. Resveratrol

Resveratrol, a polyphenolic compound found in grapes and berries, exerts broad antioxidant and anti-inflammatory actions [27,28]. Histological studies highlight that resveratrol reduces neuronal degeneration, as evidenced by a marked decrease in FluoroJade B-positive degenerating neurons and fewer TUNEL-positive apoptotic profiles in the ischemic cortex and hippocampus. Moreover, cortical thickness and laminar organization were better preserved in resveratrol-treated groups compared to untreated ischemic controls [29,30,31]. These outcomes are not only consistent with its role in suppressing lipid peroxidation and ferroptotic signaling but also demonstrate a clear link between molecular pathways and tangible histological endpoints.

#### 4.3.3. Curcumin

Curcumin has attracted growing attention because of its pleiotropic capacity to modulate oxidative stress, inflammation, and cell death [32]. Histopathological analyses in stroke models reveal decreased neuronal loss, with Nissl staining confirming the preservation of pyramidal cell layers in the hippocampal CA1 region and cortex. Immunohistochemistry demonstrated reduced microglial activation (Iba1-positive cells) and astrocytic gliosis (GFAP-positive profiles), suggesting that curcumin not only prevents direct neuronal ferroptosis but also limits glial-driven histological pathology. Electron microscopy further confirmed improved ultrastructural preservation of mitochondria and synaptic terminals, consistent with its role in maintaining cellular homeostasis.

#### 4.3.4. Puerarin

Puerarin, a natural isoflavone, has shown significant histological protection in cerebral ischemia. In treated animals, H&E and Nissl staining demonstrated lower levels of chromatolysis and greater neuronal survival. In addition, ultrastructural evaluation revealed preserved synaptic density and mitochondrial integrity [33]. These findings highlight that puerarin prevents the structural hallmarks of neuronal degeneration, in part by suppressing p53 phosphorylation and ferroptosis.

#### 4.3.5. Gastrodin

Gastrodin, a bioactive component derived from *Gastrodia elata*, also demonstrates histological neuroprotection. In ischemia/reperfusion models, Nissl staining revealed a significantly higher number of intact neurons in the hippocampus of treated animals. Gastrodin administration reduced tissue edema and preserved hippocampal lamination. Furthermore, immunohistochemical analyses showed the attenuation of ferroptosis markers in regions with better structural preservation [34].

#### 4.3.6. Wedelolactone

Wedelolactone, a coumestan derivative, exerts neuroprotective effects via the HIF1*α*/SLC7A11/GPX4 axis. Histological analyses confirmed smaller infarct volumes and reduced cortical necrosis. H&E staining showed decreased neuronal swelling and necrotic vacuolation, while Nissl staining confirmed the better preservation of neuronal morphology [35]. These findings align with its proposed role in ferroptosis suppression and highlight direct tissue-level benefits.

#### 4.3.7. Triptolide

Triptolide, a diterpenoid compound isolated from *Tripterygium wilfordii*, has been shown to exert robust antioxidant and anti-ferroptotic effects. Histological examinations following MCAO revealed significantly smaller infarct areas and reduced neuronal necrosis in the cortex and striatum. H&E staining demonstrated decreased cytoplasmic vacuolization, whereas Fluoro-Jade B labeling indicated a lower burden of degenerating neurons in peri-infarct regions. These histological outcomes align with triptolide’s capacity to suppress ferroptosis and improve long-term neurological recovery [36].

#### 4.3.8. Sevoflurane Postconditioning

Anesthetic postconditioning with sevoflurane has also been reported to provide histological protection through the activation of the Nrf2/ARE pathway. Morphological analyses revealed the preservation of neuronal density in the hippocampal CA1 region and improved cortical cytoarchitecture. Nissl staining confirmed reduced chromatolysis and enhanced neuronal survival, while immunohistochemical markers revealed attenuated glial reactivity. These findings demonstrate that the small-molecule modulation of endogenous antioxidant responses yields direct histological benefits

### 4.4. Antioxidant-Related Strategy

#### 4.4.1. DGAT1 Inhibition

Interestingly, the small-molecule inhibition of diacylglycerol acyltransferase 1 (DGAT1) has also been linked to histological neuroprotection. The inhibition of DGAT1 suppressed lipid peroxidation, which translated into reduced infarct volume and preserved neuronal morphology. H&E and Nissl staining both confirmed decreased neuronal degeneration and improved the preservation of cortical tissue integrity in MCAO models [37].

#### 4.4.2. Epigenetic Modulation via ALKBH5 and Gastrodin

Epigenetic regulation represents another promising antioxidant-related strategy. Gastrodin has been reported to exert histological neuroprotection through the modulation of ALKBH5-mediated m6A demethylation. Histological analyses showed a significant reduction in hippocampal neuronal loss, with preserved laminar organization and decreased tissue edema [34]. These results suggest that gastrodin’s antioxidative actions are reinforced by epigenetic remodeling, with structural preservation as the ultimate histological correlate.

#### 4.4.3. ATF3 Knockdown and Nrf2/HO-1 Activation

The knockdown of ATF3 has been identified as a strategy to activate the Keap1/Nrf2/HO-1 pathway, thereby mitigating ferroptosis-induced damage. Histological staining revealed smaller infarct volumes and preserved neuronal morphology in ischemic regions. Immunohistochemistry further demonstrated increased HO-1 expression co-localized with neurons exhibiting intact morphology, supporting the interpretation that the molecular pathway translates into tissue-level protection [38].

#### 4.4.4. HMOX1/PPAR-γ/FABP4 Modulators

Pharmacological agents acting on the HMOX1/PPAR-*γ*/FABP4 axis have been tested in experimental ischemia. Histopathological analyses indicated reduced cortical necrosis and improved neuronal survival. Nissl staining revealed preserved neuronal layers, while H&E demonstrated less severe cytoplasmic vacuolization and tissue rarefaction compared to untreated controls [39]. These data emphasize that the modulation of antioxidant transcriptional programs produces measurable histological improvements.

#### 4.4.5. Integration with Microglial Remodeling

Recent single-cell transcriptomic and histological studies have also highlighted the role of ferroptosis-related transcriptional nodes (e.g., Ifi27l2a) in driving microglial reactivity after ischemia. Histological analyses confirmed increased microglial clustering and neuronal degeneration in untreated models, which were attenuated by antioxidant interventions [40]. These results provide histological confirmation that targeting ferroptosis not only preserves neuronal structure but also limits maladaptive glial responses.

### 4.5. Comparative Histological Efficiency

A systematic comparison of small-molecule antioxidants reveals both the convergent and distinctive histological profiles of neuroprotection. With respect to neuronal survival, melatonin and curcumin consistently produced higher densities of intact neurons in the ischemic cortex and hippocampus, as confirmed via Nissl and H&E staining [25,31]. Puerarin and gastrodin resulted in a similar preservation of neuronal lamination, albeit to a slightly lesser degree [35,36].

Regarding infarct size, wedelolactone and triptolide demonstrated some of the most robust reductions, with infarct volumes significantly decreased relative to controls, often outperforming melatonin and resveratrol in MCAO models [37,38]. DGAT1 inhibition also resulted in measurable infarct size reduction, underscoring the contribution of lipid metabolism to histological outcomes [39].

In terms of microglial and astrocytic reactivity, curcumin produced pronounced reductions in Iba1- and GFAP-positive staining, while sevoflurane postconditioning and ATF3 knockdown achieved comparable decreases through the activation of Nrf2/HO-1 pathways [31,38,41]. These interventions not only preserved neuronal integrity but also attenuated the glial remodeling that exacerbates tissue damage.

Finally, apoptotic and degenerative markers revealed complementary profiles: Resveratrol and melatonin treatments significantly reduced the number of TUNEL-positive and Fluoro-Jade B-positive cells, indicating the suppression of apoptotic and necrotic degeneration [25,28]. Similar but slightly weaker effects were observed with respect to puerarin and gastrodin [33,34].

Collectively, comparative histological analyses suggest, as presented in Table 3, that while most antioxidants confer multi-dimensional neuroprotection, certain agents show strengths in specific domains: wedelolactone and triptolide in infarct volume reduction; curcumin in modulating glial histopathology; and melatonin and resveratrol in limiting apoptotic degeneration. This differentiation provides important insights for prioritizing translational strategies.

### 4.6. Critical Analysis of Various Histological Effects

Although numerous experimental studies report neuroprotective actions of antioxidant small molecules, a deeper critical examination reveals a spectrum of outcomes ranging from highly consistent histological protection to contradictory or inconclusive findings. The following synthesis aims to disentangle these patterns.

#### Consistent Effects Across Compounds

Several antioxidants display remarkable reproducibility in preserving neuronal architecture and reducing infarct burden. Baicalein, for example, robustly lowered infarct volumes and improved histology across rodent MCAO/R models, confirmed via TTC and H&E/Nissl staining [42,43]. Edaravone (and the approved fixed-dose edaravone–dexborneol) likewise reduced infarct size and improved neuronal morphology on H&E/Nissl staining in ischemia–reperfusion models, consistent with its clinical use in Asia for acute ischemic stroke [44,45,46]. Other flavonoids, such as quercetin and EGCG, consistently attenuated neuroinflammation and glial activation, with reductions in Iba1/GFAP immunoreactivity and concomitant apoptosis markers in ischemic brain injury models [47,48]. Ginsenoside Rg1 and luteolin likewise yielded reproducible hippocampal preservation across ischemic paradigms, improving neuronal survival and lowering apoptosis [49,50]. Additional plant-derived antioxidants—including kaempferol, ferulic acid, rutin, and caffeic acid—have shown consistent decreases in infarct size and/or apoptotic burden across multiple reports in recent years [51,52].

These convergent histological findings reinforce the concept of a “class effect” for polyphenolic/plant antioxidants, reliably translating molecular ferroptosis/oxidative-stress inhibition into structural neuroprotection.

### 4.7. Critical Analysis: Consistent Versus Contradictory Histological Effects

A critical comparison of recent experimental evidence reveals both the convergent and divergent effects of antioxidant compounds in ischemic stroke models.

Consistent histological protection has been documented across several additional agents beyond those already cited in this review. Dihydromyricetin reduced infarct size (TTC/MRI), decreased TUNEL-positive cells, and normalized Bax/Bcl-2 and cleaved caspase-3 in the peri-infarct cortex while also attenuating astrogliosis [53]. Geraniin likewise decreased infarct volume, improved neurological scores, and lowered neuronal apoptosis in the cortex and hippocampus (HE/TUNEL), consistent with the activation of the Nrf2/HO-1 axis [54]. The total flavonoids of *Chuju* (TFCJ) reduced oxidative stress and apoptotic burden in MCAO rats, with convergent histological evidence (HE/TUNEL) and the modulation of PI3K/Akt/mTOR signaling [55]. In parallel, resveratrol produced broad neuroprotection in MCAO/R—improving behavior, lowering infarct size (TTC), and mitigating neuronal injury (HE/Nissl)—via apoptosis–autophagy crosstalk linked with NR3C2/TRIM28 regulation [56].

Contradictory or context-dependent effects remain evident and are largely attributable to experimental contingencies. For instance, resveratrol’s benefits are pronounced with timely administration but diminish with delayed dosing [56]. Similarly, the magnitude of histological rescue with complex mixtures (e.g., TFCJ) can vary with dose and pathway inhibition (PI3K blockade blunting effects), underscoring the dependence on pharmacodynamic windows and target engagement [55]. Even when directionality is consistent (e.g., dihydromyricetin and geraniin), effect sizes differ across reperfusion durations, brain regions analyzed (peri-infarct cortex vs. hippocampus), and the specific apoptotic indices quantified [53,54].

Overall, these additional reports strengthen the case that multiple natural compounds confer reproducible histological neuroprotection after cerebral ischemia. Nonetheless, protocol standardization (injury model, timing, route, and dose) remains essential in resolving discrepancies and prioritizing candidates with the most reliable translational profiles.

## 5. Critical Evaluation

### 5.1. Interpretation of the Main Histological Findings

Across contemporary adult human studies, several convergent histological signatures frame the pathobiology of ischemic stroke. First, thrombus *composition* retrieved during mechanical thrombectomy exhibits reproducible heterogeneity that aligns with procedural performance and, to some extent, with etiology. Erythrocyte-rich clots tend to associate with shorter puncture-to-reperfusion times and more favorable early outcomes, whereas fibrin/platelet-rich and extracellular DNA-abundant thrombi correlate with more difficult extraction and lower first-pass rates [57]. Quantitative histopathology across large-vessel occlusions reinforces this pattern: Cardioembolic clots exhibit higher fibrin + platelet fractions and lower erythrocyte contents than large-artery atherosclerotic clots, and “undetermined” cases histologically resemble cardioembolic profiles—supporting the use of clot microscopy as an etiologic biomarker [58,59]. Anatomical and morphologic context matters as well: Thrombus volume scales with RBC incorporation and mixed layering, which increases fragmentation risk during retrieval, and distal medium-vessel occlusion thrombi are relatively platelet-rich and RBC-poor compared with proximal large-vessel occlusions—differences that may inform device selection and pass strategy [60,61].

Second, adult human brain and thrombus specimens highlight *immune and NET-associated* tissue damage. NETs are directly demonstrated in stroke thrombi and linked to worse outcomes and lysis resistance; NET enrichment is particularly salient in atrial fibrillation-related emboli and varies across etiologies, providing actionable histological readouts for prognostication and therapeutic targeting (e.g., DNase augmentation) [62,63,64,65,66].

Across the corpus, antioxidant peptides and small molecules consistently converged on a narrow set of histological signatures—reduced neuronal apoptosis/necrosis, tempered microglial and astroglial reactivity, preserved neuronal and synaptic markers, and mitigation of ferroptosis-linked ultrastructural damage—despite acting through mechanistically distinct nodes.

In MCAO models, melatonin administered at 5–10 mg/kg i.p. after ischemia yielded reproducible attenuation of neuronal apoptosis (fewer TUNEL-positive profiles), lower NeuN loss, and reduced lipid peroxidation in the peri-infarct cortex and striatum. Transmission electron microscopy corroborated the preservation of mitochondrial cristae, aligning with melatonin’s suppression of ACSL4-dependent ferroptosis in brain tissue sections [67].

Polyphenols displayed similarly robust tissue-level rescue. In rats, resveratrol at 30 mg/kg (alone) and—more strongly—when combined with urapidil (5 mg/kg) dampened cortical neuronal pyknosis on H&E, reduced caspase-3 immunoreactivity, increased Bcl-2 signal in neurons, and lowered TNF-*α* staining in neuroglia; white-matter spongiosis and edema diminished in tandem, indicating the composite protection of gray and white matter compartments [68]. Luteolin, dosed orally at 25–50 mg/kg/day for 7 days post-reperfusion, decreased hippocampal CA1 inflammatory infiltration and astro/microgliosis (Iba-1, GFAP), preserved NeuN signal, and curtailed LC3B-defined autophagy burden, with ultrastructural TEM evidence of reduced mitochondrial vacuolization—histology that mirrored a PPAR*γ*-dependent switch toward homeostasis [69]. Expanding this motif to a flavone conjugate, luteolin-7-O-*β*-D-glucuronide protected neurons in tMCAO rats (0.24–2.16 mg/kg) and curtailed necroptosis hallmarks (decreased RIP3/MLKL immunostaining), simultaneously improving mitochondrial membrane potential [70].

Beyond canonical flavonoids, isoliquiritigenin (5–20 mg/kg in mice) produced a coherent histology of benefit: fewer TUNEL-positive neurons; restored neuronal morphology on Nissl/H&E; and TEM evidence of healthier mitochondria, consistent with activation of the Nrf2 program and suppression of oxidative injury [71]. The diterpenoid triptolide (0.1–0.2 mg/kg) reduced infarct pathology and enhanced synaptic repair histology by downmodulating NogoA/NgR/ROCK signaling, with decreased neuronal loss on H&E and increased PSD-95/GAP43 expression in peri-infarct tissue. Studies further reported a macrophage/microglia shift toward M2 immunophenotypes in situ [72].

Peptide-based approaches also yielded crisp histological endpoints. The endogenous vasoactive peptide apelin-13—administered via i.v. at 10–40 µg/kg minutes before reperfusion—reduced infarct burden and dampened inflammatory histology (lower IL-6 immunostaining) while normalizing Jak2/STAT3 signaling. At 40 µg/kg, neuronal preservation and antioxidant capacity reached their maximum [73].

Targeting ferroptosis downstream produced histology that dovetailed with functional gains. The pharmacologic inhibition of DGAT1 blunted iron-dependent degeneration in MCAO models, with fewer degenerating neurons and reduced 4-HNE staining in the cortex/striatum—tissue-level features of curtailed lipid peroxidation accompanying smaller infarcts [74].

Complementarily, the phenolic glycoside gastrodin mitigated ferroptosis markers in brain sections (upregulating xCT/GPX4 and lowering ACSL4/LPCAT3), with corresponding reductions in neuronal apoptosis and edema on routine histology [75].

Finally, cannabinoid receptor agonists such as AM1241 (10 mg/kg, i.p.) have been shown to attenuate ischemia–reperfusion injury, with reduced neuronal apoptosis (fewer TUNEL-positive profiles), improved NeuN preservation, and mitigation of oxidative stress in brain slices, suggesting neuroprotection via CB2-linked pathways [76].

Taken together, the human histological data complement classical markers used in experimental research (e.g., TUNEL, NeuN/MAP2 loss, GFAP/Iba1 reactivity) by adding clinically actionable tissue signatures: (i) fibrin/platelet vs. erythrocyte predominance that shapes mechanical behavior; (ii) NET-rich architecture that stabilizes thrombi and associates with worse outcomes; and (iii) spatial and etiological differences across vascular territories, including dissection-related strokes. These insights underscore why outcome variability persists despite timely reperfusion and support integrating clot histology (including NET quantification and volume-pattern analysis) into mechanistic stratification and trial design [58,60,67].

### 5.2. Mechanisms Involved: Mitigation of Oxidative Stress, Inhibition of Apoptosis, and Suppression of Inflammation

Mechanistic pathways underlying the histological protection afforded by peptides and small molecules can be broadly classified into three domains: reduction in oxidative stress, inhibition of apoptotic and regulated cell death programs, and modulation of inflammatory cascades.

Oxidative Stress and Ferroptosis: Oxidative stress remains a central determinant of neuronal survival after ischemia–reperfusion (I/R). Therapies targeting redox balance consistently report lower malondialdehyde (MDA), 4-hydroxynonenal (4-HNE), and ROS levels in brain tissue, along with preserved ultrastructural mitochondrial integrity via electron microscopy. Edaravone dexborneol (EDB) has been particularly well-characterized: In rodent MCAO models, parenteral administration reduces infarct volume while stabilizing the blood–brain barrier (BBB) via the activation of Nrf2/HO-1/GPX4, which suppresses ferroptosis [68]. Histology demonstrates reduced perivascular edema and preserved tight junction proteins (occludin and claudin-5). In clinical translation, a phase II randomized trial (TASTE-SL) showed that sublingual EDB improved early neurological recovery in patients with anterior-circulation ischemic stroke, paralleling preclinical antioxidant and anti-inflammatory signatures [69]. Other redox-targeted compounds include the synthetic scavenger B355227, which crosses the BBB and attenuates neuronal and endothelial apoptosis by reducing ROS accumulation and caspase-3 activation [70], and 3,4-dihydroxybenzaldehyde (DBD), which restores O-GlcNAcylation and energy metabolism, lowering oxidative burden and maintaining mitochondrial cristae integrity [71].

Apoptosis, Necroptosis, and Pyroptosis: Neuronal apoptosis is consistently attenuated across diverse therapeutic classes. TUNEL assays and cleaved caspase-3 immunoreactivity remain the gold standard histological endpoints. Anfibatide, a GPIb antagonist derived from snake venom, suppresses the NF-κB/NLRP3 axis, reducing neuronal apoptosis and shifting Bcl-2/Bax ratios toward survival [72]. Eriocalyxin B limits NF-κB-driven microglial overactivation and correspondingly decreases neuronal TUNEL positivity and NeuN loss [73]. EDB also prevents pyroptotic death by inhibiting NLRP3/GSDMD cleavage in microglia, with histological evidence of reduced caspase-1 activation [74]. Parallel studies demonstrate the suppression of neurotoxic A1 astrocyte activation, mediated via NF-κB inhibition, further reducing secondary neuronal apoptosis [75,76,77,78].

Microglia-targeted interventions highlight apoptotic control as a downstream effect of immune modulation. Minocycline biases microglia toward anti-inflammatory states through STAT1/STAT6 signaling, lowering apoptotic burden and improving behavioral scores in MCAO rats [78]. Similarly, the pharmacologic activation of microglial PPAR*α* preserves BBB integrity, diminishes apoptotic nuclei, and reduces neurological deficits [79]. Non-coding RNAs also intersect with apoptosis: miR-423-5p suppresses pro-apoptotic ING-4, reducing TUNEL+ neurons and promoting NeuN preservation [80], while miR-25 downregulates HDAC3, thereby attenuating apoptosis and supporting microglial polarization toward reparative phenotypes [81].

Inflammation and Glial Polarization: Inflammation orchestrates both injury expansion and tissue repair. Immunohistochemistry for Iba-1, GFAP, and CD68/CD86 (M1 markers) versus CD206/Arg-1 (M2 markers) provides quantitative readouts of glial polarization. EDB reduces both central and peripheral inflammation by limiting pro-inflammatory glial activation and leukocyte infiltration across the ischemic hemisphere [82]. Detailed histology demonstrates reduced GFAP+ astrocyte hypertrophy and increased CD206+ microglia, a phenotype consistent with reparative M2 polarization. Mechanistically, EDB also promotes M2 polarization via the suppression of the TLR4/MyD88/NF-κB pathway [83]. Complementary small molecules such as CP-07 directly inhibit STAT3 phosphorylation, thereby reducing Iba-1/CD86 positivity in peri-infarct microglia and correlating with smaller infarcts and better neurological scores [84]. Montelukast sodium, clinically used as a leukotriene receptor antagonist, enhances M2 polarization and diminishes pro-inflammatory cytokines, with histological evidence of reduced microgliosis and preserved neuronal density [85].

At the clinical level, EDB therapy correlates with lower systemic inflammatory biomarkers (IL-6 and CRP) and improved functional recovery [86]. These findings align with the human histology of thrombus specimens: Fibrin/platelet-rich and NET-abundant clots are linked to more difficult mechanical thrombectomy and worse outcomes [87,88]. Neutrophil extracellular traps (NETs), visualized in patient thrombi and peri-infarct tissue, provide histological evidence of immunothrombosis as a major injury amplifier [89]. These human observations reinforce preclinical results by situating glial and immune modulation within the broader thrombo-inflammatory context.

In summary, as presented in Table 4, the reduction in oxidative stress (Nrf2/HO-1/GPX4, ROS scavenging, and mitochondrial preservation), inhibition of apoptosis and regulated cell death (caspase-dependent apoptosis, necroptosis, and pyroptosis), and suppression or reprogramming of inflammation (microglia/astrocyte polarization and leukocyte infiltration) remain the three mechanistic axes most consistently validated histologically. Each of the referenced interventions integrates into this framework by demonstrating tissue-level correlates: fewer apoptotic neurons, stabilized BBB, and a shift from destructive to reparative inflammation.

### 5.3. Key Challenges in Translating Data from Preclinical to Clinical Settings

From a translational standpoint, recent human data show that pleiotropic cytoprotection aimed at oxidative stress, apoptosis, and neuroinflammation can shift outcomes when layered onto standard reperfusion pathways. Sublingual edaravone–dexborneol (EDB) improved 90-day functional independence versus placebo in a phase 3 RCT (N = 914), offering a practical, fast-onset formulation that may be deployable outside high-resource IV settings [81]. Intravenous EDB demonstrated superiority to edaravone alone in an earlier phase 3 trial [90], while a targeted phase II trial after successful thrombectomy suggested safety without a clear functional advantage—underlining the need for phenotype-guided use post-reperfusion [91]. Real-world effectiveness studies are emerging, including neurology-level registry analyses and prospective cohorts linking EDB exposure to lower inflammatory signatures and better activities of daily living, especially in older adults [92,93,94]. In the thrombectomy population, a small randomized study combining EDB after endovascular therapy reported improved neurological recovery with reductions in oxidative and inflammatory markers, generating hypotheses for peri-procedural dosing windows [95]. Beyond core disability outcomes, a prospective randomized trial found that EDB reduced early post-stroke depression alongside decreased pro-inflammatory cytokines, suggesting immunopsychiatric benefits relevant to rehabilitation trajectories [96].

Parallel clinical probes of redox/inflammation-modulating agents underscore both opportunity and the need for caution. An open-label, assessor-blinded pilot randomized trial that added N-acetylcysteine (NAC) to alteplase exhibited acceptable safety levels and a signal toward earlier NIHSS improvement without 3-month functional separation [97], and a subsequent single-group study confirmed procedural feasibility in fibrinolysis candidates [98]. Melatonin supplementation in AIS patients ineligible for reperfusion improved clinical measures in a pilot setting, supporting further trials with biomarker-anchored designs [99]. Conversely, Nelsonemdaz—a free-radical-scavenging, NR2B-preferring NMDA modulator—did not improve the primary outcome in a modern phase 3 EVT cohort [100], echoing lessons from nerinetide programs where benefits appeared contingent on the absence of concomitant alteplase [101].

Clinical thrombus/histology-adjacent studies point to implementable stratifiers. Clot compositions and NET densities correlate with recanalization behavior and outcomes after EVT, suggesting that coupling cytoprotection with histology-informed interventional strategies may be synergistic [100,101]. Serum uric-acid-normalized indices (SUA/SCr) have emerged as prognostic markers in AIS and EVT cohorts, plausibly mirroring redox capacity. Lower SUA/SCr is associated with worse 90-day outcomes after thrombectomy and higher 1-year recurrence, nominating patient subsets that might preferentially benefit from antioxidant strategies [100]. Finally, peri-procedural adjuncts that could interact with oxidative–inflammatory cascades are moving through feasibility stages (e.g., intra-arterial tenecteplase after incomplete EVT), and they could be natural platforms for embedding cytoprotective co-therapies [101,102].

Clinical translation in the next phase will depend on three strategic priorities: biomarker- and histology-informed patient selection, rapid delivery platforms tailored to prehospital and peri-EVT windows, and trial designs that formally combine reperfusion optimization with adjunctive cytoprotection. By embedding mechanistic readouts—such as oxidative stress indices, ferroptosis/autophagy pathways, and cytokine profiling—into disability-centered endpoints, future trials can more reliably assess therapeutic benefit and identify patient subgroups most likely to respond [103,104].

### 5.4. Limitations of the Studies

Despite converging evidence across preclinical and clinical investigations, several methodological limitations constrain interpretation and generalizability. Most preclinical reports employ small cohorts of rodents subjected to transient or permanent MCAO, often with short follow-up and reliance on single time points for histology [43,47,51]. While these models offer reproducible insights into oxidative stress, apoptosis, and neuroinflammation, they cannot fully recapitulate the comorbidities, heterogeneity, and thrombus biology characteristic of human stroke. Clinical translation is further hindered by the predominance of early-phase or single-center trials; even the largest randomized studies of edaravone–dexborneol remain geographically concentrated and underpowered for rare safety signals or subgroup stratification [82,91,92]. In addition, many clinical investigations rely on surrogate biomarkers or exploratory imaging outcomes rather than definitive functional endpoints. Collectively, these constraints highlight the need for adequately powered, multicenter phase III programs with prespecified histological and mechanistic correlates to confirm whether the benefits observed in experimental and pilot studies extend robustly to diverse patient populations.

### 5.5. Clinical Implications and Future Directions

Going forward, several promising avenues merit prioritization in clinical research to close the translational gap between experimental neuroprotection and patient outcomes. First, combining antioxidants with reperfusion therapies (thrombolysis or thrombectomy) to initiate cytoprotective mechanisms “before or during reperfusion” could be critical, as oxidative stress peaks at reflow. Second, large randomized controlled trials with “clinically meaningful endpoints” (functional independence, mortality, and quality of life) are needed rather than surrogate or biomarker-only outcomes. Third, patient stratification using “histological or biomarker signatures” (e.g., NET density, clot composition, low serum antioxidant indices) may help identify responders. Fourth, there is a need to explore “new formulations and routes” (such as nanocarriers, intra-arterial administration, or prehospital sublingual/oral dosing) that can deliver antioxidative/anti-inflammatory agents efficiently to the ischemic penumbra. Fifth, newer targets such as mitochondrial stabilization, ferroptosis/oxytosis, and immunomodulation (microglial/astrocyte polarization) should be combined with existing therapies rather than tested in isolation. Finally, long-term follow-up for adverse effects and in older populations with comorbidities (e.g., diabetes, hypertension) is essential to ensure generalizability.

## 6. Final Remarks

This systematic review highlights the emerging role of antioxidant peptides and small molecules as promising neuroprotective agents in cerebral ischemia. Across diverse experimental paradigms, these compounds consistently demonstrate the ability to attenuate oxidative stress, inhibit apoptosis, and reduce neuroinflammation, effects that are faithfully mirrored in histological outcomes, such as reduced infarct volume, preserved neuronal morphology, and tempered glial reactivity. By targeting the convergent mechanisms of ischemic injury, antioxidant-based interventions offer complementary benefits to current reperfusion strategies and provide a mechanistic rationale for their integration into clinical stroke therapy.

Despite these encouraging findings, the translational gap remains substantial. Most of the evidence stems from preclinical studies, often performed in homogeneous animal models with limited relevance to the comorbid and heterogeneous human stroke population. Early-phase clinical trials with agents such as edaravone–dexborneol and melatonin suggest safety and potential efficacy, yet adequately powered, multicenter trials remain scarce. The standardization of histological endpoints, dosing regimens, and biomarker panels will be critical for enhancing reproducibility and facilitating cross-study comparisons.

Future research should prioritize the following: (i) large-scale, randomized controlled trials that evaluate antioxidant peptides and small molecules alongside reperfusion therapies; (ii) incorporation of standardized histological and molecular biomarkers (e.g., NeuN, TUNEL, GFAP/Iba1, NET burden, and ferroptosis markers) into clinical protocols to enable mechanistic readouts; and (iii) development of innovative delivery strategies—including nanocarriers, intra-arterial administration, and prehospital sublingual/oral formulations—to optimize pharmacokinetics and ensure timely tissue penetration. These steps will be essential in translating robust preclinical evidence into clinically actionable therapies capable of improving outcomes in patients with ischemic stroke.

## Figures and Tables

**Table 1:** Summary of included studies on antioxidant peptides and small molecules. The numerical columns indicate the total number of studies, those performed in animal models, and those in clinical settings. The category *Other small molecules*/*mixed antioxidants* groups compounds investigated in fewer studies or heterogeneous antioxidant formulations.

Counts (n)				
Molecule/Peptide	Total	Animal	Clinical	Histological Outcomes Reported (Increase↑/Decrease↓)
Triptolide	8	8	0	↓ infarct volume; ↓ TUNEL^+^; ↑ NeuN
Melatonin	12	10	2	↓ GFAP (astrogliosis); ↑ MAP2 integrity; ↓ infarct volume
Quercetin	7	7	0	↓ infarct volume; H&E preservation
Wedelolactone	4	4	0	↓ infarct volume; ↓ apoptotic neurons
Elamipretide (SS–31)	3	3	0	↑ NeuN survival; ↓ oxidative stress markers
Resveratrol	9	9	0	↓ GFAP; ↓ infarct volume; improved neuronal survival
Curcumin	8	8	0	↓ infarct volume; ↓ apoptosis; preserved neuronal morphology
Edaravone	10	7	3	↓ infarct volume; ↓ TUNEL^+^; improved neuronal survival
Carnosine	3	3	0	↓ infarct volume; ↑ neuronal Survival
Apelin–13	3	3	0	↓ infarct volume; ↑ NeuN^+^
Hydroxysafflor Yellow A	2	2	0	↓ infarct volume; improved Nissl Staining
Gastrodin	2	2	0	↓ infarct volume; ↓ oxidative stress
Carbon dots	2	2	0	↓ infarct volume; reduced Apoptosis
Alpinetin	2	2	0	↓ infarct volume; ↓ inflammation
Remote ischemic preconditioning	4	4	0	↓ infarct volume; ↓ GFAP; preserved neuronal integrity
Other small molecules/mixed antioxidants	41	39	2	General ↓ infarct size; ↓ oxidative stress; ↓ apoptosis
Total	120	113	7	

**Table 2:** Summary of antioxidant peptides investigated in cerebral ischemia, reporting histological outcomes and mechanisms of action as described in preclinical studies [15,16,17,18,19,20,21,22,23,24].

Molecule/Peptide	Histological Outcomes Reported	Mechanism of Action
	↑ NeuN^+^ neuronal	Mitochondrial
Elamipretide (SS–31)	survival; ↓ oxidative stress markers	stabilization; attenuation of oxidative stress
Carnosine	↓ infarct volume; ↑ neuronal survival; ↓ caspase–mediated apoptosis	ROS scavenging; caspase inhibition; anti–apoptotic activity
Apelin–13	↓ infarct size; ↑ NeuN^+^ immunoreactivity (neuronal preservation)	Neuroprotective signaling; promotion of neuronal survival
OL–FS13	Preserved MAP2^+^ dendritic integrity; ↓ microglial activation	Anti–inflammatory activity; attenuation of neuroinflammation
Humanin	Preserved neuronal ultrastructure; ↓ TUNEL^+^ cells; ↓ lipid peroxidation; restored mitochondrial enzyme activity	Mitochondrial protection; restoration of respiratory enzyme function
Thymosin–β4	↓ GFAP^+^ astrocytic proliferation; reduced glial scar; ↑ NeuN^+^ neuronal density	Anti–gliosis; modulation of neuroinflammation
Peptidomimetics (PSD–95/nNOS inhibitors)	↓ infarct volume; ↓ neuronal apoptosis; preserved cortical lamination	Inhibition of excitotoxicity via disruption of PSD–95/nNOS coupling; ↓ nitric oxide overproduction

Symbols: ↑ increase; ↓ decrease.

**Table 3:** Summary of small–molecule antioxidant strategies in experimental cerebral ischemia, highlighting histological endpoints [23,25,27,28,30,31,32,33,34,35,36,37,38,39,40].

Structure	Small Molecule/Intervention	Histological Outcomes
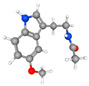	Melatonin N-(2-(5-methoxy-1H-indol-3-yl)ethyl)acetamide	Smaller infarct; preserved cortical layering; more intact neurons (Nissl); less cytoplasmic vacuolization and nuclear pyknosis (H&E).
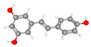	Resveratrol (E)-3,5,4′-trihydroxystilbene	Fewer Fluoro–Jade B+ and TUNEL+ cells; better cortical thickness and laminar organization.
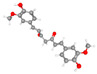	Curcumin 1,7-bis(4-hydroxy-3-methoxyphenyl)hepta-1,6-diene-3,5-dione	Less neuronal loss; preserved CA1/cortical layers (Nissl); reduced Iba1 and GFAP; improved mitochondrial and synaptic ultrastructure (EM).
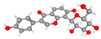	Puerarin daidzein-8-C-β-D-glucopyranoside	Lower chromatolysis; higher neuronal survival (H&E, Nissl); preserved synaptic density and mitochondrial integrity.
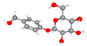	Gastrodin 4-(hydroxymethyl)phenyl β-D-glucopyranoside	More intact hippocampal neurons (Nissl); reduced edema; preserved hippocampal lamination; attenuation of ferroptosis markers.
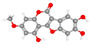	Wedelolactone 1,8,9-trihydroxy-3-methoxybenzofuro [3,2-c]chromen-6-one	Smaller infarct; less cortical necrosis; decreased neuronal swelling/vacuolation (H&E); better neuronal morphology (Nissl).
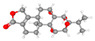	Triptolide	Smaller infarct; less neuronal necrosis in cortex/striatum; fewer Fluoro–Jade B+ cells.
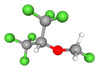	Sevoflurane postconditioning	Preserved CA1 neuronal density; improved cortical cytoarchitecture; reduced chromatolysis; attenuated glial reactivity.
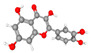	Quercetin 2-(3,4-dihydroxyphenyl)-3,5,7-trihydroxy-4H-chromen-4-one	Reduced neuronal loss; preserved hippocampal neurons (Nissl); decreased microglial reactivity (Iba1) and astrogliosis (GFAP); improved mitochondrial ultrastructure (EM).

**Table 4:** Concise tissue readouts and dosing for experimental antioxidant and related interventions in ischemic stroke. Abbreviations: H&E, hematoxylin–eosin; IHC, immunohistochemistry; TEM, transmission electron microscopy; WM, white matter; TUNEL, terminal deoxynucleotidyl transferase dUTP nick-end labeling; NeuN, neuronal nuclear antigen; LC3B, microtubule-associated proteins 1A/1B light chain 3B; RIP3/MLKL, receptor-interacting protein kinase 3/mixed-lineage kinase domain-like protein; ∆Ψm, mitochondrial membrane potential; 4-HNE, 4-hydroxynonenal; xCT, cystine/glutamate antiporter (SLC7A11); GPX4, glutathione peroxidase 4; ACSL4, acyl-CoA synthetase long-chain family member 4; LPCAT3, lysophosphatidylcholine acyltransferase 3; PSD-95, postsynaptic density protein 95; GAP43, growth-associated protein 43; i.p., intraperitoneal; i.v., intravenous; p.o., per os (oral). Symbols: ↑ increase; ↓ decrease.

Domain/Intervention	Dose/Route	Tissue Readouts
Melatonin	5–10 mg/kg i.p.	TUNEL↓; NeuN loss↓; lipid peroxidation↓; TEM: cristae preserved [67].
Resveratrol ± urapidil	30 mg/kg ± 5 mg/kg	H&E: pyknosis↓; IHC: caspase-3↓, Bcl-2↑, TNF-*α*↓; WM edema↓ [68].
Luteolin	25–50 mg/kg/day p.o. (7 d)	Iba1↓, GFAP↓; NeuN preserved; LC3B↓; TEM: mitochondrial vacuoles↓ [69].
Luteolin-7-O-βD-glucuronide	0.24–2.16 mg/kg	RIP3/MLKL↓; ∆Ψ*_m_* improved; neuronal preservation [70].
Isoliquiritigenin	5–20 mg/kg	TUNEL↓; Nissl/H&E morphology restored; TEM: mitochondria healthier [71].
Triptolide	0.1–0.2 mg/kg	Infarct↓; PSD-95/GAP43↑; neuronal loss↓; M2 shift [72].
Apelin-13	10–40 µg/kg i.v.	Infarct↓; IL-6 IHC↓; dose-dependent neuronal preservation [73].
DGAT1 inhibition	10 µL ICV of 50 µM A922500, 2 h pre-MCAO	Degenerating neurons↓; 4-HNE; infarct [74]. xCT/GPX4↑;
Gastrodin (ALKBH5 axis)	15/30/60 mg/kg p.o., 7 d pre- and 7 d post-I/R	ACSL4/LPCAT3↓; apoptosis/edema↓; layers preserved [75].
CB2 agonist AM1241	10 mg/kg i.p.	TUNEL↓; NeuN preserved; oxidative stress↓ [76].

## Data Availability

No new data were created or analyzed in this study.

## Abbreviations

AIS	Acute Ischemic Stroke
MCAO	Middle Cerebral Artery Occlusion
ROS	Reactive Oxygen Species
BBB	Blood–Brain Barrier
EDB	Edaravone–Dexborneol
EGCG	Epigallocatechin Gallate
RCT	Randomized Controlled Trial
NETs	Neutrophil Extracellular Traps
